# A rare case of retroperitoneal ganglioneuroma in adult: diagnostic and management challenges

**DOI:** 10.1093/jscr/rjag098

**Published:** 2026-04-15

**Authors:** Olivia Camilleri, Chahaya Gauci, David Wilson, Kirk Austin

**Affiliations:** Department of Colorectal Surgery, Royal Prince Alfred Hospital, 50 Missenden Road, Camperdown NSW 2050, Australia; Department of Colorectal Surgery, Royal Prince Alfred Hospital, 50 Missenden Road, Camperdown NSW 2050, Australia; Department of Colorectal Surgery, Royal Prince Alfred Hospital, 50 Missenden Road, Camperdown NSW 2050, Australia; Department of Neurosurgery, Royal Prince Alfred Hospital, 50 Missenden Road, Camperdown NSW 2050, Australia; Department of Colorectal Surgery, Royal Prince Alfred Hospital, 50 Missenden Road, Camperdown NSW 2050, Australia

**Keywords:** retroperitoneal ganglioneuroma, rare, diagnostic delay, radiology, multidisciplinary care, surgical intervention

## Abstract

Retroperitoneal ganglioneuromas (RGNs) are a rare form of neuroblastic tumour. Despite being benign they pose significant clinical challenges due to their non-specific symptoms, variable radiographic features and technically difficult resection. We present a case of RGN in a male in his early twenties, with a 4-year history of multiple symptoms including bone and joint pains in his chest, abdomen and right hip. Blood results were normal, and magnetic resonance imaging revealed a large mass close to L4/L5 nerve roots, overlying the right ureter and inferior vena cava. The mass was resected in a joint case with colorectal and neurosurgical teams and histopathology confirmed ganglioneuroma. This case report contributes valuable insight into the clinical presentation, diagnostic workup, and surgical management of RGN. We demonstrate the importance of considering this rare diagnosis in the work up of patients with non-specific symptoms and present considerations for surgical resection, advocating for a multidisciplinary approach.

## Introduction

Ganglioneuroma is a benign and rare type of sympathetic nervous system tumour, arising from neural crest cells [1]. Ganglioneuromas typically occur in patients aged 20-35 years [2] and primary sites correspond to the migration pattern of neural crest cells in embryological development. Ganglioneuromas are seen most in the posterior mediastinum, retroperitoneum and adrenal gland [3]. RGNs are considered rare, with a reported incidence of one per million population [4], and constituting only 0.72% to 1.6% of primary retroperitoneal tumours [3].

The nature of RGNs makes diagnosis difficult. RGNs often cause nonspecific symptoms, growing indolently until large enough to cause symptoms of mass effect. Imaging findings also vary, contributing to diagnostic difficulty. On computerized tomography (CT) ganglioneuromas are commonly solid and well circumscribed with mild post contrast enhancement but can also be cystic or calcified with mixed density. On magnetic resonance imaging (MRI) they are most commonly hypointense on T1 weighted images and hyperintense on T2 but have also been described as heterogeneous, which could be mistaken for pathologies including sarcoma [5, 6]. Although malignant transformation to neuroblastoma is rare, biopsy cannot capture the tumours entire histological profile and may miss malignant cells [7]. Therefore, definitive diagnosis relies on histopathology of the entire surgical resected specimen [2, 7]. Microscopically, ganglioneuromas are characterized by mature ganglion cells amongst schwannian stroma [1]. Surgical resection can be difficult due to challenging access to the retroperitoneum and proximity to major vascular structures.

## Case report

**Figure 1:**
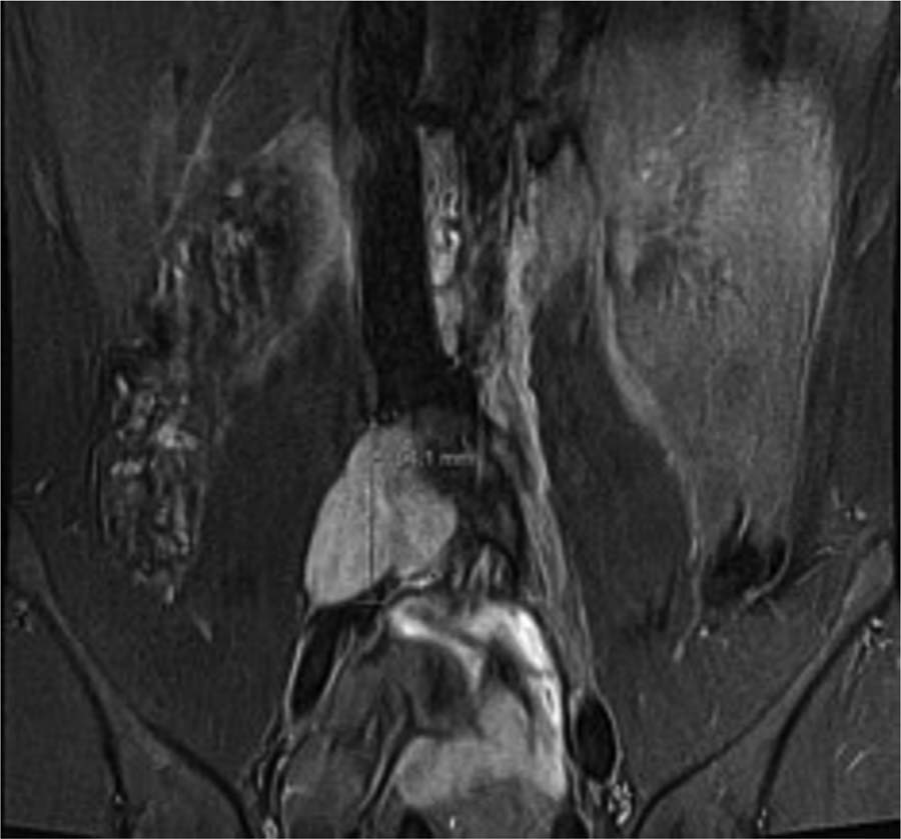
Coronal short TI inversion recovery (STIR) showing hypointense lobulated lesion from L4-L5 to L5-S1 level, medial to the right psoas muscle.

**Figure 2:**
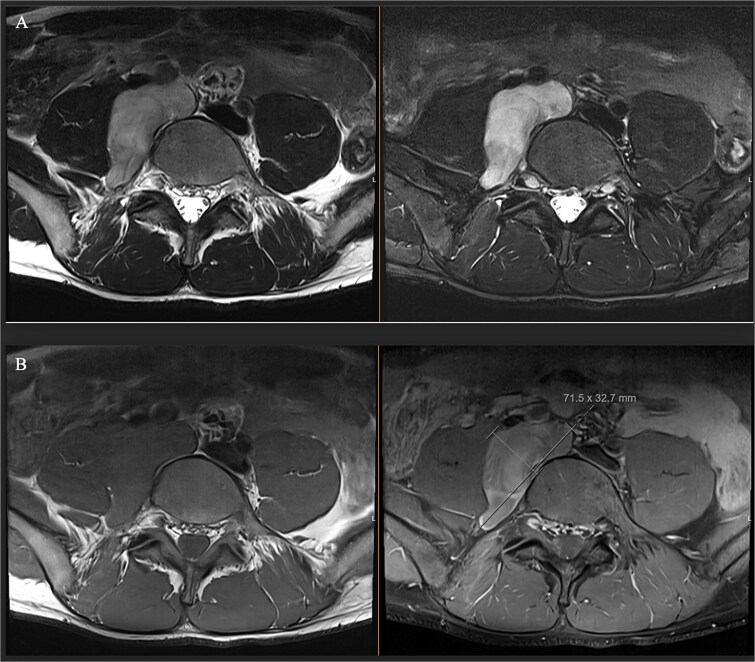
(A) Axial T1 MRI: Left) pre-contrast, right) post contrast demonstrating mild contrast enhancement. (B) Axial T2 MRI: Left) axial T2, right) axial T2 with fat saturation showing hypertense lesion displacing iliac vessels and psoas.

A male in their early twenties presented with a 4-year history of a multitude of symptoms including bone and joint pains in his chest, abdomen and lesser right hip pain. He was seen by an osteopathy and physiotherapist and then referred to a GP who performed plain x-rays of bilateral hips and an abdominal ultrasound, which were normal. His symptoms persisted, and back MRI showed a lobulated mass in the right paraspinal region extending from the L4-L5 to the L5-S1 level, medial to the psoas muscle (Fig. 1), displacing the iliac vessels anteriorly and psoas laterally without involvement of exiting nerves or lumbar plexus (Fig. 2).

On examination his vitals were normal, he appeared well with his abdomen soft and non-tender. He had no past medical history, no regular medications and unremarkable family history. He is a non-smoker, denied alcohol use and is physically active in his work and hobbies which were restricted by his symptoms. Routine bloods were unremarkable. Alpha-fetoprotein, beta human chorionic gonadotropin, serum metanephrine were within normal limits.

The patient was discussed at the sarcoma multidisciplinary team meeting, with recommendation for positron emission tomography (PET) and CT guided biopsy. Cytology sample was limited but showed smears of low cellularity with occasional groups of spindle cells embedded within stroma, and blank cells with little atypia. Histopathology from core biopsy showed tissue compromising of bland wavy spindle cells arranged into loose fascicles characteristic of Schwann cells. Large ganglion cells were present with round nucleic, prominent nucleoli and abundant cytoplasm, indicating mature ganglion cells. PET demonstrated minimal glucose avidity within the lesion only.

Ganglioneuroma was the principal differential. Bloods results were reassuring against a malignant neuroblastic, mixed germ cell tumour or lymphoma. MRI demonstrated common features suggestive but not exclusive to ganglioneuroma including T1 hypointensity and T2 hyperintensity, and mild contrast enhancement [5] (Figs 1 and 2). Differentials included a nerve sheath tumour however there was no definitive associated exiting nerve or lumbar plexus involvement on MRI. Minimal glucose avidity on PET also pointed towards a benign process. The histopathological findings of mature ganglion cells mixed with Schwan cells are the hallmark of ganglioneuroma and important for distinguishing this benign tumour from neural crest derived tumours [8].

**Figure 3:**
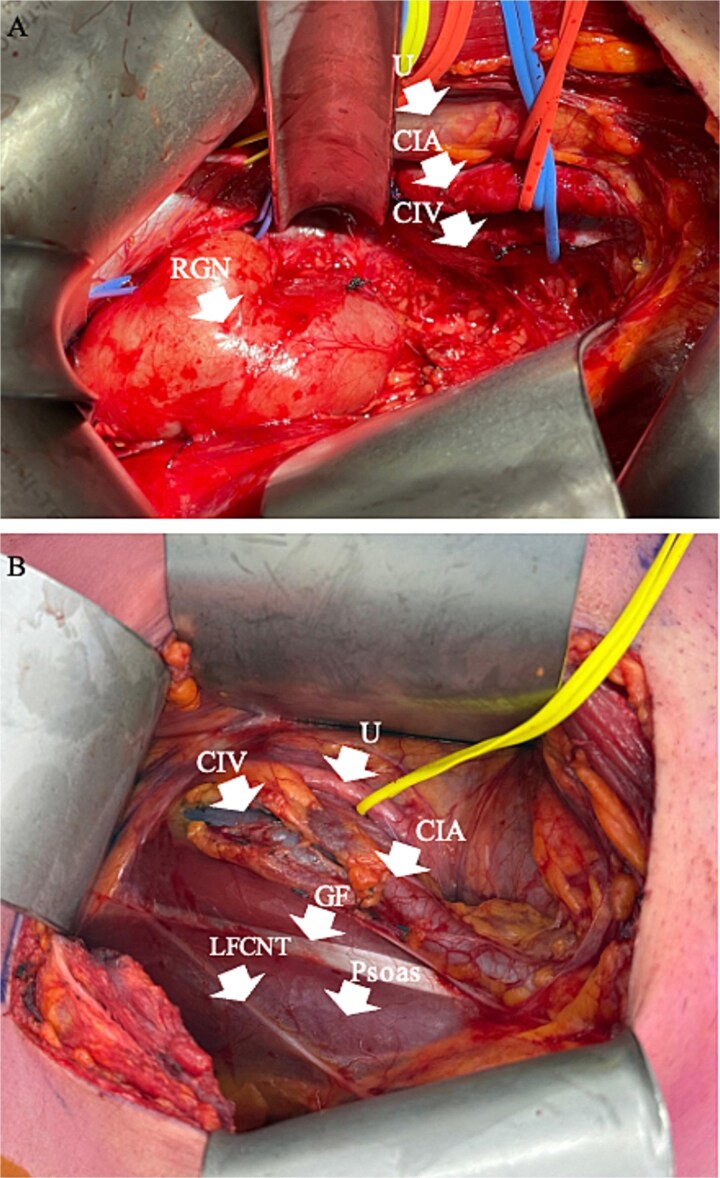
Intraoperative pictures pre and post dissection of RGN. (A) Intraoperative picture of retroperitoneal dissection, with arrows pointing to RGN and isolated structures. (B) Intraoperative picture post resection of RGN. RGN = retroperitoneal ganglioneuroma, U = ureter, CIA = common iliac artery, CIV = common iliac vein, GF = genitofemoral nerve, LFCNT = lateral femoral cutaneous nerve of the thigh.

Joint operation was performed by colorectal and neurosurgery teams. A right Rutherford Morrison incision was made, and the retroperitoneum accessed via an extraperitoneal approach. Major vascular structures were mediatized along with the ureter (Fig. 3A) to gain vascular control, including dissection of right common iliac vessels to the external iliac junction and mobilization of the IVC by ligating lumbar tributaries at L4 and L5 vertebral levels. The medial border of the right psoas muscle was mobilised off the lateral wall of the ganglioneuroma. Medially, the vertebral bodies of L4 and L5 were exposed and L4 and L5 nerve roots identified and protected. The ganglioneuroma was then dissected free from the retroperitoneum (Fig. 3B), with L4 and L5 nerve roots identified and dissected off the lesion under microscopic guidance. A 19 French Blake drain was inserted into the retroperitoneal space.

Histopathology was consistent with ganglioneuroma being composed of a dominant Schwannian spindle cell stroma (>90% of the tumour S100- positive) with scattered ganglion cells. The resection was complete with no evidence of malignancy.

The patient had an unremarkable post operative period. Drain was removed on Day 6 and he was discharged on Day 7. He was reviewed by the colorectal consultant at 3 weeks and is doing well. He will be followed up again at 9 weeks followed by repeat MRI at 6 months and 1 year.

## Discussion

As demonstrated by this case, RGNs present considerable diagnostic challenges. The non-specific presentation, indistinct imaging patterns, and biopsy limited by sampling error highlight the importance of understanding and considering RGN in similar patients [7]. Management should involve evaluation of malignant risk, symptom burden and surgical risk. Transformation of ganglioneuroma to neuroblastoma is extremely rare, with biopsy proven ganglioneuromas stable on 2 year follow up being unlikely to continue to growth significantly or undergo malignant transformation [7]^.^ Therefore, observation may be appropriate for asymptomatic patients, surgically irresectable tumours or those with significant co-morbidities making surgery high risk [7]. Our patient was well, and his preference being for operative management due to symptom burden. Appropriate counselling on the risk of neurovascular injury was important and we adopted a multidisciplinary approach to limit potential morbidity in the face of a technically demanding resection. Complete excision by marginal resection is considered curative and whilst there are no clear guidelines on ganglioneuroma follow up, surveillance should be determined by post-operative course, residual tumour and symptom burden [7].

## References

[1] Ikegaki HSN . Peripheral neuroblastic tumors. Enzinger and Weiss's Soft Tissue Tumors 2020;28:991–1008.

[2] Kordeni K, Chardalias L, Pantiora E et al. Retroperitoneal ganglioneuroma presenting as lower back pain. J Surg Case Rep 2022;2022:rjac082. 10.1093/jscr/rjac082 PMC9015710 35444793

[3] Xiao J, Zhao Z, Li B et al. Primary retroperitoneal ganglioneuroma: a retrospective cohort study of 32 patients. Front Surg 2021;8:642451. 10.3389/fsurg.2021.642451 PMC8176303 34095202

[4] Shekhar S, Vakharia R, Van Hoven AM. Retroperitoneal ganglioneuroma presenting as symptomatic nephrolithiasis in a young adult: a zebra among horses. AACE Clin Case Rep 2018;4:140–2.

[5] Cai J, Zeng Y, Zheng H et al. Retroperitoneal ganglioneuroma in children: CT and MRI features with histologic correlation. Eur J Radiol 2010;75:315–20. 10.1016/j.ejrad.2010.05.040 20580177

[6] Luo L, Zheng X, Tao KZ et al. Imaging analysis of ganglioneuroma and quantitative analysis of paraspinal ganglioneuroma. Med Sci Monit 2019;25:5263–71. 10.12659/MSM.916792 31306406 PMC6647925

[7] Noh S, Nessim C, Keung EZ et al. Retrospective analysis of retroperitoneal-abdominal-pelvic ganglioneuromas: an internati-onal study by the transatlantic Australasian retroperitoneal sarcoma working group (TARPSWG). Ann Surg 2023;278:267–73. 10.1097/SLA.0000000000005625 35866666 PMC10191524

[8] Shimada H, Ambros IM, Dehner LP et al. Terminology and morphologic criteria of neuroblastic tumors: recommendations by the international Neuroblastoma pathology committee. Cancer. 1999;86:349–63. 10421272

